# Sexual harassment-abuse and psychotherapy: the strenght of therapeutic relathionship

**DOI:** 10.1192/j.eurpsy.2022.1925

**Published:** 2022-09-01

**Authors:** C. Moutafi, I. Kalafataki

**Affiliations:** Child’s Mental Health Center, Department Foradolescents, ATHENS, Greece

**Keywords:** sexual abuse, sexual harassment, therapeutic relationship, mental health

## Abstract

**Introduction:**

The term trauma comes from the ancient Greek word “titrosko” than means perforate. Sexual harassment and abuse of a person during childhood is an important risk factor for mental trauma.

**Objectives:**

Present the impact of sexual harassment and abuse in the mental health of adolescents and the imprortance of therapeutic relationship.

**Methods:**

From the literature review the child needs love which is demostrated with tenderness. The adult (perpetrator) with a disorder responds to the child’s tenderness with the language of passion. The immature Ego of the child is not strong enough to deal with the adult behavior and this causes anxiety, helplessness, confusion and guilt about the relationship with the adult. During the psychotherapeutic process, 4 main protagonists emerge : the victim, the perpetrator, an absent mother and an omnipotent savior.

**Results:**

Mental trauma can adversely affect the development of the neurobiological system resulting in difficulty coping with stressful events. Untreated trauma can lead to serious psychopathology such as anxiety disorders , depressive disorder, personality disorders, addictions. The creation of a therapeutic relationship, understanding the adolescent and his family potential, the recognition and treatment of transference-countertransference phenomena and the existence of a clinical setting that acts as a restraint mechanism could contribute to the therapy of mental trauma.

**Conclusions:**

The Therapeutic Department for Adolescents could be an environment to contain, process and transform the painful into pleasant emotions, as well as aiming the authenticity of the person with a history of sexual harassment and abuse.

**Disclosure:**

No significant relationships.

